# Simultaneously Monitoring and Reducing Nε-Carboxymethyl-Lysine and 5-Hydroxymethylfurfural Contents During Soy Sauce Production and Consumption

**DOI:** 10.3390/foods14142437

**Published:** 2025-07-10

**Authors:** Yongtai Wu, Bei Hu, Yuxin Wen, Zuowei Xiao, Lin Li, Xia Zhang, Zhenhui Zhang, Bing Li

**Affiliations:** 1 Guangdong Provincial Key Laboratory of Green Processing of Natural Products and Product Safety, School of Food Science and Engineering, Engineering Research Center of Starch and Plant Protein Deep Processing Ministry of Education, South China University of Technology, 381 Wushan Road, Tianhe District, Guangzhou 510640, China; yontawu@gmail.com (Y.W.); hub@dgut.edu.cn (B.H.); yuxinwen1997@163.com (Y.W.); 003831@hnucm.edu.cn (Z.X.); cexzhang@scut.edu.cn (X.Z.); 2 School of Life and Health Technology, Dongguan University of Technology, College Road 1, Dongguan 523808, China; lilin@dgut.edu.cn; 3 School of Pharmacy, Hunan Engineering and Technology Research Center for Health Products and Life Science, Hunan University of Chinese Medicine, Changsha 410208, China; 4 College of Food and Biological Engineering, Henan University of Animal Husbandry and Economy, Zhengzhou 450046, China

**Keywords:** Maillard reaction, soy sauce, Nε-carboxymethyl-lysine, 5-hydroxymethylfurfural, production, consumption

## Abstract

Soy sauce (SS) is one of the most popular condiments in the world. However, Nε-carboxymethyl-lysine (CML) and 5-hydroxymethylfurfural (5-HMF), harmful Maillard reaction products, are present in SS. Worse still, their primary sources in SS production remain unclear, and their contents increase during the consumption of heated SS. In this study, CML and 5-HMF were simultaneously monitored, and thermal treatment and the addition of natural product were used to simultaneously reduce their contents during SS production and consumption. During SS production, CML and 5-HMF primarily originated from the raw materials used in SS production, Maillard reactions during fermentation, and the addition of food additives. Also, CML and 5-HMF were simultaneously found in commercial light soy sauce, dark soy sauce, and infant SS, and thermal treatment could increase their contents. Fortunately, additional thermal treatment of semi-finished SS (especially raw sauce and rude light SS) and appropriate concentrations of (−)-epicatechin (100 μM) and ascorbic acid (5 μM), respectively, added to SS for direct and heated consumption, could simultaneously reduce the CML and 5-HMF contents. This study highlights the presence of CML and 5-HMF in SS and proposes practical methods to simultaneously minimize their contents during production and consumption.

## 1. Introduction

Soy sauce (SS) is one of the most popular traditional condiments in the world due to its saltiness and umami taste and is expected to have a global market size of USD 13,600 million with a compound annual growth rate of 3.6% from 2025 to 2031 [1,2]. SS is traditionally produced by fermenting soybean pulp, wheat, brine, and *Aspergillus oryzae* or *Aspergillus sojae* molds for several months [3,4]. During fermentation, protein and starch from soybeans and wheat are gradually hydrolyzed into amino acids and reducing sugar, respectively [1]. Then, the Maillard reaction inevitably proceeds during fermentation and sterilization. On the one hand, the Maillard reaction products endow SS with its pronounced flavor and taste; on the other hand, some harmful Maillard reaction products, such as Nε-carboxymethyl-lysine (CML) and 5-hydroxymethylfurfural (5-HMF), are produced concomitantly [5,6]. Li et al. [7] detected the CML contents of a total of 12 brands of Chinese and Japanese SS products, with a maximum content of 988.79 μg/mL of soy sauce. Worse still, thermal treatments, such as sterilization and cooking, promote the formation of CML and 5-HMF and increase human intake of CML and 5-HMF from SS [8]. However, SS production involves multiple processes, such as mixing, pre-fermentation, fermentation, and sterilization, which can cause different changes in the CML and 5-HMF contents in semi-finished and finished SS. Hence, the main sources of CML and 5-HMF in SS production remain unclear, making it difficult to implement effective methods to reduce their contents during SS production.

In the Maillard reaction, CML and 5-HMF are concurrently generated. CML is a typical advanced glycation end product (AGE), while 5-HMF is a furfural compound. Both are associated with the hazards of the Maillard reaction [9,10]. CML has been proven to increase oxidative stress levels, disrupt the gut flora, cause cellular metabolic disorders, lead to brain aging, and have a subacute toxicity profile in the nervous system [11,12,13,14]. 5-HMF is associated with toxicity and mutagenicity [9] and has been found to have carcinogenic properties and cause DNA damage, kidney cytotoxicity, and liver mutagenicity [15]. Most importantly, CML and 5-HMF are predominantly present in the free state in SS, which facilitates their absorption in the gastrointestinal tract to undermine our health [16,17]. Moreover, the combined effect of the different hazards of the Maillard reaction could aggravate their perniciousness [18]. Therefore, the simultaneous intake of CML and 5-HMF in SS should attract more attention. Simultaneous monitoring of the CML and 5-HMF contents during SS production can facilitate the implementation of targeted measures to reduce their contents. Moreover, during SS consumption, this monitoring approach can assist consumers in identifying healthy SS for a healthy lifestyle.

Shortening the reaction time and lowering the reaction temperature are common practices to effectively limit the degree of the Maillard reaction, consequently inhibiting CML and 5-HMF formation [19]. However, this reduces the original color and flavor substances in foods. On the contrary, recent studies have found that prolonging the Maillard reaction can reduce CML and 5-HMF when the Maillard reaction proceeds to a certain extent. Extending the reaction time and increasing the reaction temperature can reduce the CML contents in foods or Maillard reaction model systems [20,21]. 5-HMF is unstable and reacts with other amino compounds, such as ammonia and amino acids, to transform into harmless compounds after further reaction processes [22,23]. Therefore, prolonging the Maillard reaction with extra thermal treatment may be another way to reduce the CML and 5-HMF contents and optimize the SS production process.

Moreover, polyphenols can inhibit the formation of reactive oxygen species, Schiff bases, and carbonyl compounds, which are the precursors of CML and 5-HMF [24]. With the development and healthy demands of the SS industry, natural products, especially tea extracts, have been introduced to cater to consumers’ demands. (−)-Epicatechin (EC) and (−)-epigallocatechin gallate (EGCG) are the main polyphenols in tea extract, and they can eliminate CML and 5-HMF. Li et al. [25,26] showed that catechin quinones derived from oxidized EC or EGCG could react with CML by a Michael addition or Schiff base addition. Also, EC could react with 5-HMF to form a dimer or trimer. EGCG inhibited 5-HMF formation by reducing 3-deoxuglucosone [27,28]. Therefore, EC and EGCG are used to simultaneously reduce the contents of CML and 5-HMF in SS.

In this study, simultaneous detection of CML and 5-HMF was adopted to improve detection efficiency during SS production. In addition, to clarify the main sources of CML and 5-HMF during SS production and the exposure contents during SS consumption, free CML and 5-HMF were simultaneously determined in raw materials, semi-finished products, and commercial SS products. Moreover, to reduce the CML and 5-HMF contents in SS, thermal treatment was performed on raw materials and semi-finished products, and EC, EGCG, and VC were added to SS for different methods of consumption. This study aims to simultaneously monitor the contents of CML and HMF during the production and consumption of soy sauce, providing a reliable theoretical basis for the implementation of measures to reduce the contents of CML and HMF in the SS industry. Meanwhile, this study also provides two methods to reduce the content of CML and HMF in soy sauce from SS production and consumption, which will reduce the intake of CML and 5-HMF from SS and provide effective means to enhance the safety and quality of SS.

## 2. Materials and Methods

### 2.1. Materials

The raw materials of SS were purchased from a seasoning company in Guangdong, China. Six kinds of SS were bought from the local market. Nε-carboxymethyl-lysine (purity > 99%) was from TRC Corporation (Toronto, Canada). 5-hydroxymethylfurfural (purity > 98%) was purchased from Shanghai Yuanye BioTechnology Co., Ltd. (Shanghai, China). (−)-epicatechin (EC) (purity ≥ 97%), (−)-epigallocatechin gallate (EGCG) (purity ≥ 98%), and ascorbic acid (VC) (purity ≥ 98%) were purchased from Aladdin Reagent Co., Ltd. (Shanghai, China). Chromatographic pure methanol and formic acid were purchased from Thermo Fisher Scientific Co., Ltd. (Waltham, MA, USA) and Merck KGaA (Darmstadt, Germany), respectively.

### 2.2. Sample Preparation

**Figure 1:**
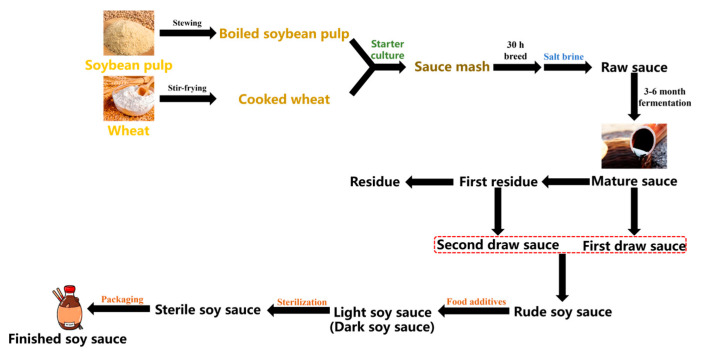
SS production process (LSS is light soy sauce, DSS is dark soy sauce, and the red frame means that first draw sauce and second draw sauce are mixed together.).

Raw materials and semi-finished SS were provided by the local SS industry (Guangdong, China), and the schematic diagram of SS preparation is shown in Figure 1. Cooked wheat and boiled soybean pulp were mixed with starter culture (including *Aspergillus oryzae* and *Aspergillus sojae* molds) to obtain sauce mash for 30 h of pre-fermentation at 45 °C. Cold saline solution by mass fraction of 18% was added to the fermentation cylinder, and raw sauce was obtained. After 3 months of naturally sealed fermentation in an open-air environment, the mature sauce was obtained, including the liquid first draw sauce and the first solid residue. A second fermentation was subsequently carried out on the first residue, and a second draw sauce was obtained after another 3 months of fermentation. Rude SS was obtained from the mixture of the first draw sauce and second draw sauce. According to the product requirements, various food additives, such as flavor amino acids, sweeteners, and caramel pigment, were added to obtain modified light soy sauce (LSS) or modified dark soy sauce (DSS). After sterilization and packaging, the production of SS was completed.

In the simulation experiment of SS consumption, six commercial SSs were mixed in equal proportions to eliminate the individual differences. Different concentrations (5 μM, 10 μM, 100 μM, and 1000 μM) of EC, EGCG, or VC were dissolved in the mixed SS and stored at room temperature or in a thermal environment as described in 2.4. VC was used as a positive control. The sample without adding EC, EGCG, and VC was used as a control.

### 2.3. Detection of Physicochemical Indexes

Water content, reducing sugar content, soluble salt-free solid content, and free amino acid (FAA) contents were determined according to previous research [29,30,31]. Total nitrogen and protein contents were measured by the Kjeldahl method using an automatic Kjeldahl apparatus (FOSS Kjelte 8400, Copenhagen, Denmark) and calculated using Equations (1) and (2), respectively.

(1) $$A=\left(A3-A1\right)*6.25,$$

(2) $$A3=\frac{A2}{M}$$

where A is protein contents (g/100g), A1 is FAA contents (g/100 g), A2 is nitrogen content (mg N), A3 is total nitrogen (g/100 g), M is the mass of tested SS (g), and 6.25 is the reduction coefficient that 1 mg of nitrogen is equal to 6.25 mg of protein.

### 2.4. Thermal Treatment

Samples (1 g) were dissolved in distilled water (10 mL) and allowed to stand for 30 min before centrifugation (7000 rpm, 4 °C, 15 min). The supernatant liquid was transferred into a sealed serum bottle. After heating at 80 °C for 30 min, the solution was stored at −20 °C until the free CML and 5-HMF contents were detected. The samples were kept at room temperature for 30 min as a control.

### 2.5. Simultaneous Detection of Free CML and 5-HMF

Free CML and 5-HMF contents was determined simultaneously according to our previous research with some modifications [32]. In detail, SS (1 g) was dissolved in distilled water (10 mL) and then stirred at room temperature for 30 min before centrifugation (7000 r/min, 4 °C, 15 min). Prior to HPLC-MS/MS analysis, the supernatant liquid (1 mL) was fractionated on a PolyClean X-MCX solid phase extraction column. Impurities were removed twice with distilled water (10 mL), and the target was eluted twice with 10% ammonia-methanol (10 mL) and collected. The eluate was dried under nitrogen before being dissolved in 10% methanol for HPLC-MS/MS analysis. A total of 0.01–2 μg/mL of mixed CML and 5-HMF standards was used to depict the calibration curve.

Quantitative analysis of CML and 5-HMF was performed according to the method of Nguyen [21] with some modifications. The separation of CML and 5-HMF was performed on a Proshell 120 SB-C18 (50 mm × 3.0 mm, 2.7 μm, Agilent, Shanghai, China). The mobile phase consisted of 0.1% (*v/v*) aqueous formic acid solution (A) and methanol (B). The flow was 90% solvent A, maintained at 0.5 mL/min for 5 min, and detected by triple quadrupole mass spectrometry. Positive electrospray ionization with multiple reaction monitoring (MRM) was used to simultaneously monitor parent and fragment ions of CML and 5-HMF. The capillary temperature, atomizing gas pressure, and capillary voltage were 300 °C, 0.1 MPa, and 4.0 kV, respectively. For CML and 5-HMF, the respective transitions of *m/z* 205–130 and *m/z* 127–81 were used for qualitative analysis, while *m/z* 205–84 and *m/z* 127–109 were used for quantitative analysis, respectively.

### 2.6. Statistical Analysis

All measurements were performed in triplicate, and the values are expressed as mean ± standard error and calculated using SPSS 20.0. Analysis of variance (ANOVA) indicates significant differences within and between groups (*p* < 0.05), with uppercase indicating significant differences between groups and lowercase indicating significant differences within the groups.

## 3. Results and Discussions

### 3.1. The CML and 5-HMF Contents During SS Production

**Figure 2:**
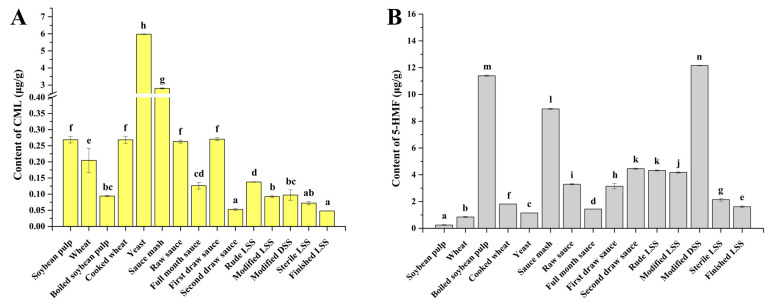
The contents of CML (**A**) and 5-HMF (**B**) in raw materials and semi-finished SS (the lowercase letters indicate significant differences among different samples).

Several steps in SS production would lead to the formation and accumulation of CML and 5-HMF, which are still unclear. To identify the main sources of CML and 5-HMF during SS production, the contents of CML and 5-HMF in raw materials and semi-finished SS were simultaneously determined (Figure 2). Among raw materials, starter culture and boiled soybean pulp contained the highest level of CML (5.98 ± 0.02 μg/g) and 5-HMF (11.39 ± 0.05 μg/g), respectively. Among semi-finished SS, sauce mash contained the highest content of CML (2.81 ± 0.03 μg/g SS) and the second highest 5-HMF (8.92 ± 0.05 μg/g SS), and modified DSS contained the highest content of 5-HMF (12.16 ± 0.02 μg/g). Based on the different contents of CML and 5-HMF in raw materials and semi-finished SS, as well as the production process of SS, the changes in the contents of CML and HMF during the SS production are inferred. The starter culture had been pre-fermented for several days to breed fermented colonies before SS fermentation [4]. During this period, numerous reducing sugars were released and reacted with amino compounds to generate glyoxal. Subsequently, glyoxal reacts with the released lysine to produce CML via the Maillard reaction [33]. In addition, 5-HMF could accumulate in food under thermal treatment [34]. High water activity inhibited the degradation of 5-HMF and the reaction between 5-HMF and amino acids [23,35,36], resulting in the accumulation of a large amount of 5-HMF in boiled soybean pulp. After mixing the raw materials, all the CML and 5-HMF derived from raw materials were transferred to the sauce mash, and most of them were gradually released into the sauce after the addition of salt brine and fermentation. It is worth noting that the combined amount of CML or 5-HMF in the first draw sauce and second draw sauce was greater than that in the raw sauce, which indicated that CML and 5-HMF were continuously produced by the Maillard reaction during fermentation. Moreover, DSS needed more caramel pigment to provide color, and caramel pigment contained a large amount of 5-HMF [37], so modified DSS had the most 5-HMF.

Therefore, the accumulation of CML and 5-HMF during SS production began with the starter culture and boiled soybean pulp. Then, CML and 5-HMF were transferred to the sauce mash after mixing with the original fermentation substrate. Subsequently, CML and 5-HMF were further produced by the Maillard reaction during fermentation. Finally, CML and 5-HMF were introduced by the addition of food additives, specifically caramel pigment containing 5-HMF.

**Figure A1:**
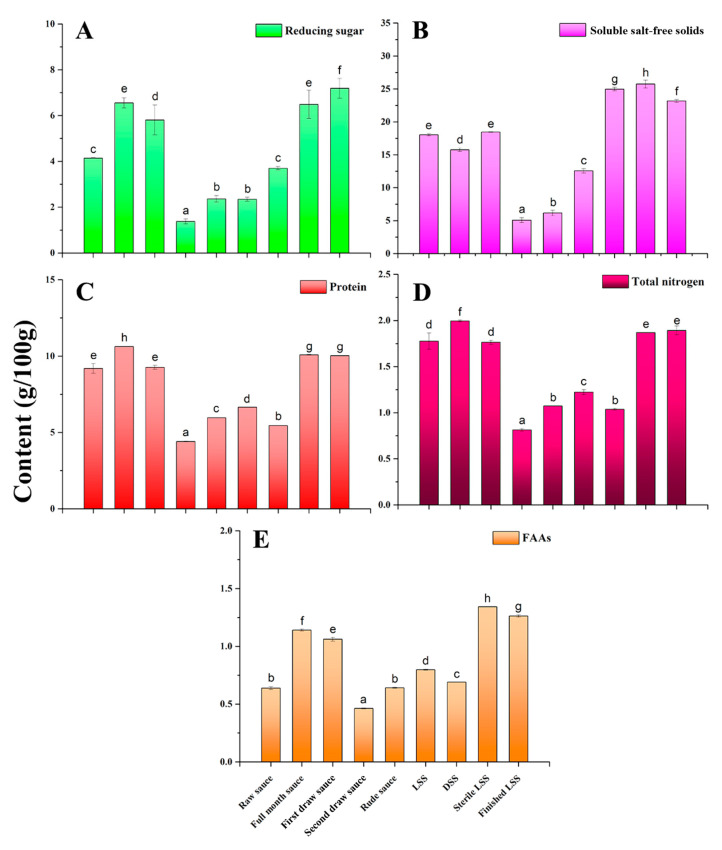
The contents of reducing sugar (**A**), soluble salt-free solids (SSS, (**B**)), protein (**C**), total nitrogen (**D**), and free amino acids (FAAs, (**E**)) in semi-finished SS (the lowercase letters indicate significant differences among different samples).

To reveal the impact of physicochemical indexes on the contents of CML and 5-HMF during SS production, the contents of basic substances in semi-finished SS were measured (Figure A1), and Spearman correlation analysis was performed between the physicochemical indexes and the contents of CML or 5-HMF (Table 1). The results showed that the content of CML was affected by the soluble salt-free solids in the reaction system, rather than by reducing sugars, FAAs, amino acid nitrogen, or total nitrogen. The formation of CML was related to multiple factors. In the Maillard reaction, reducing sugars are catalytically cleaved by amino compounds to form glyoxal, which then reacts with lysine to produce CML. It indicated that the increase in amino compounds and the decrease in lysine did not promote the formation of CML [33]. Additionally, different kinds of metal ions could promote or inhibit the Maillard reaction to generate CML [38]. All these substances belong to soluble salt-free solids. Moreover, SS is a complex liquid system, containing the soluble salt-free solids that affect the generation of CML. Significantly, the soluble salt-free solids in SS are mainly composed of reducing sugar and proteins (Table 1). Hence, the more solid substances there are in soy sauce, the more CML will be produced, but it will also be interfered with by other substances, such as metal ions in SS, at the same time. Compared to CML, 5-HMF had an extremely significant negative correlation with reducing sugars, protein, FAAs, and total nitrogen. 5-HMF was primarily produced by the hydrolysis of monosaccharides [39,40], and amino compounds could react with 5-HMF to generate harmless compounds [23,36]. Therefore, less reducing sugar indicates that more 5-HMF is generated, and more protein, FAAs, and other amino compounds mean that 5-HMF is more easily formed.

### 3.2. The Contents of CML and 5-HMF in Commercial SS

**Figure 3:**
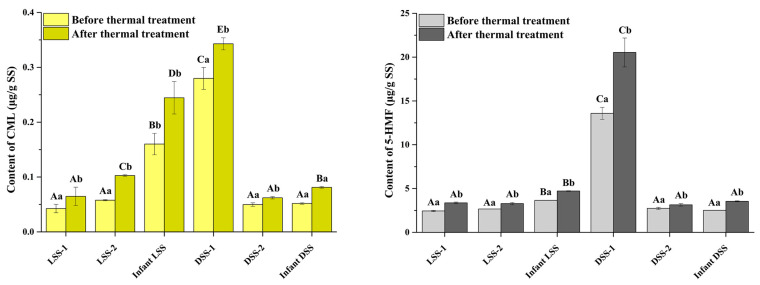
The contents of CML and 5-HMF in two brands of three kinds of commercial SS before and after thermal treatment (the uppercase letters indicate significant differences among different samples, and the lowercase letters indicate significant differences before and after thermal treatment).

SS is not only eaten directly but also consumed after hot cooking such as steaming, boiling, frying, and deep-frying. Therefore, to clarify the exposure levels of CML and 5-HMF during SS consumption, their contents were simultaneously determined before and after thermal treatment. Thermal treatment was carried out by heating at 80 °C for 30 min, and commercial LSS, DSS, and infant SS were purchased from local markets for detection (Figure 3). Different SS contained different contents of CML and 5-HMF, which could be attributed to their different raw materials and production parameters. SS contained 0.04–0.27 μg/g of CML and 2.44–13.58 μg/g of 5-HMF, and thermal treatment could increase their contents (0.06–0.34 μg/g of CML and 3.14–20.53 μg/g of 5-HMF). After fermentation and the addition of food additives, commercial SSs contain abundant amino acids and reducing sugars, and additional thermal treatment could promote the Maillard reaction to produce CML and 5-HMF [19]. Additionally, according to the China National Food Industry Association report, Chinese residents consume about 8.9 g of SS per day, which means that the maximum daily intake of CML and 5-HMF from SS in direct consumption can reach 2.40 μg and 120.86 μg, respectively. Worse still, thermal treatment during daily cooking can significantly increase these values, increasing CML and 5-HMF contents by more than 50%. When CML and 5-HMF enter the body along with SS, they will accumulate in various organs within the body, posing a hidden danger to physical health, such as a series of chronic diseases [41].

### 3.3. The Effect of Thermal Treatment on CML and 5-HMF Contents During SS Production Process

**Figure 4:**
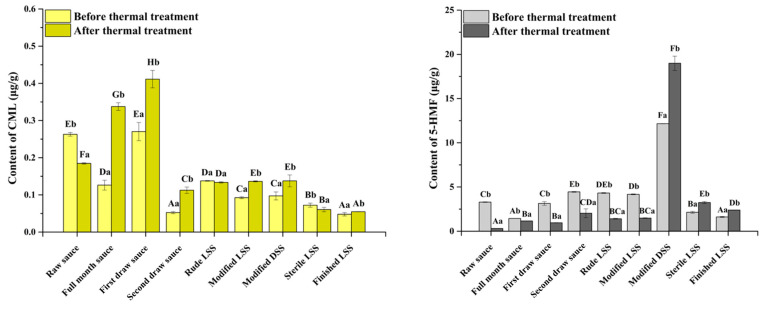
The change of CML (**left**) and 5-HMF (**right**) contents in semi-finished SS with the effect of thermal treatment (the uppercase letters indicate significant differences among different samples, and the lowercase letters indicate significant differences before and after thermal treatment).

Although the limitation of the Maillard reaction can inhibit the formation of CML and 5-HMF, it affects the quality of SS [19]. It has been found that deepening the degree of the Maillard reaction could reduce the CML and 5-HMF contents [21,36,42]. If it is feasible in semi-finished SS, it will be easier and more convenient to reduce the CML and 5-HMF contents in SS. Hence, thermal treatment was carried out on semi-finished SS at different stages of the SS production process (Figure 4). Thermal treatment could significantly eliminate the CML in raw sauce, rude LSS, and sterile LSS, as well as 5-HMF in most of the semi-finished SS except modified DSS, sterile LSS, and finished LSS. On the one hand, the fermentation and the addition of food additives enriched free amino acids and reducing sugars in the sauce, which promoted the Maillard reaction and the formation of CML and 5-HMF during thermal treatment. On the other hand, thermal treatment could eliminate CML by promoting the reaction of CML with other Maillard reaction products to form melanoidins, and 5-HMF by combining with other amino compounds [21,36,42]. It indicated that the formation and degradation of CML and 5-HMF could reach a balance, and thermal treatment had different effects on different semi-finished SS. Therefore, thermal treatment could be performed in raw sauce and rude LSS to reduce the CML and 5-HMF contents during SS production, simultaneously. However, thermal treatment could inactivate the flora in raw sauce and stop the subsequent fermentation. Therefore, thermal treatment performed in rude LSS was more in line with the SS production process, although it only reduced the 5-HMF content in the sauce.

### 3.4. The Effect of EC, EGEG, and VC on CML and 5-HMF Contents in SS Consumption

**Figure 5:**
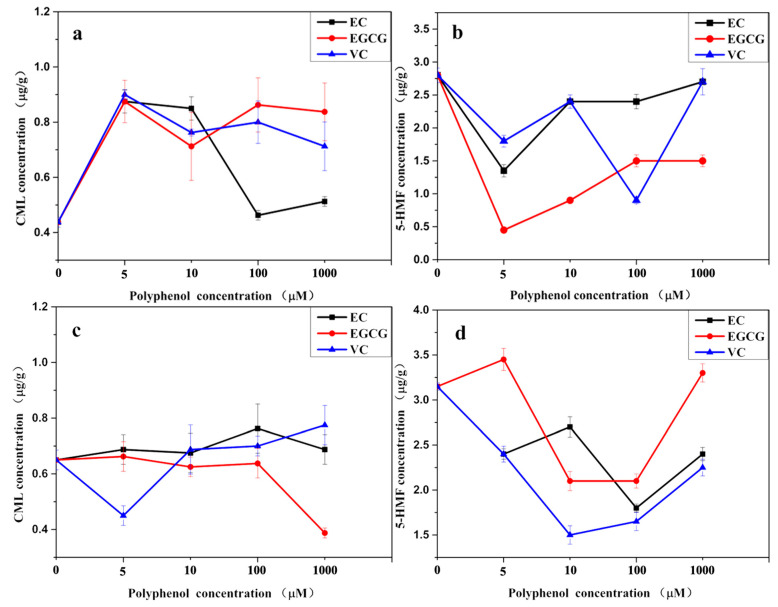
Effect of different concentrations of inhibitors (EC, EGCG, and VC) on CML and 5-HMF production ((**a**,**b**) represent direct consumption; (**c**,**d**) represent heated consumption).

CML and 5-HMF are found in SS, and heating SS can promote their formation [43,44]. The formation of CML and 5-HMF involves the free radical chain reaction and requires carbonyl compounds as precursors [45]. Polyphenols can eliminate free radicals and carbonyl compounds to inhibit the formation of CML and 5-HMF [24]. EC and EGCG can also eliminate CML and 5-HMF by scavenging [25,46,47]. Therefore, EC and EGCG were used to reduce the CML and 5-HMF contents in SS, and their effects on SS consumption were investigated, while VC was used as a positive control. Six commercial SSs were mixed in equal proportions for investigation to eliminate the individual differences in SS (Figure 5).

In direct consumption of SS, the addition of EC, EGCG, and VC increased the CML content, while a high concentration (≥100 μM) of EC did not (Figure 5a). The deprotonation of the phenolic hydroxyl groups on EC could generate free hydrogen radicals and phenolic radicals [48]. The free hydrogen radicals could attack the unstable free radicals, while the phenolic radicals could capture reactive carbonyl species (RCS), therefore inhibiting CML formation [49]. However, the absence of thermal treatment limited the generation of free radicals and RCS from the Maillard reaction, and the hydrogen radicals released by the low concentration of EC instead promote the generation of CML through the free radical reaction, as does EGCG [24]. Also, although VC could act as a radical and carbonyl scavenger to inhibit the formation of CML, it was also one of the precursors of CML [50], for which the addition of VC increased the CML content in SS direct consumption. Additionally, low concentration (5 μM) of EC and EGCG could significantly reduce the 5-HMF content in SS direct consumption, while VC required 100 μM (Figure 5b). It indicated that EC and EGCG could effectively capture 5-HMF at room temperature; however, high concentrations could cause them to aggregate and affect their ability to capture 5-HMF. The A ring in EC could react with the carbonyl in 5-HMF to form a dimer or trimer [25,26]. EGCG, which has an A ring like the EC, could bind 5-HMF as well, but with less stability than EC [51]. The ability of VC to eliminate free radicals could inhibit the formation of 5-HMF, but the degradation of VC could promote the formation of 5-HMF [52]. Therefore, the excessively high concentrations of VC would facilitate this reaction process.

Upon heating SS to simulate cooking conditions, EC, low concentrations of EGCG (≤100 μM), and high concentrations of VC (≥10 μM) had no significant effect on CML; however, 1000 μM of EGCG and 5 μM of VC significantly reduced the CML content (Figure 5c). Also, thermal treatment increased the optimal concentration of EC (100 μM) and EGCG (10 μM) to reduce 5-HMF content, but decreased the VC (Figure 5d). It was possible that EC and EGCG were inactivated by the thermal treatment or reacted with other active compounds at low concentrations, for which EC and EGCG required higher concentrations to exert the best activity in reducing 5-HMF, but excessively high concentrations could cause them to aggregate and affect their activity. Moreover, VC at the proper concentration could reduce the CML and 5-HMF contents. However, higher concentration could provide more precursors for CML 5-HMF, and also promote the Maillard reaction by ascorbic free radicals during thermal treatment to generate more CML and 5-HMF [50,53,54].

Considering the simultaneous limitation of CML and 5-HMF in SS consumption, it would be better to add 100 μM of EC to SS direct consumption, and 5 μM of VC was added to SS heated consumption.

## 4. Conclusions

CML and 5-HMF were inevitably generated through the Maillard reaction during SS production and consumption, and their combined effect could aggravate the perniciousness. Therefore, the contents of CML and 5-HMF are simultaneously detected, and additional thermal processing and the addition of exogenous substances are carried out to simultaneously reduce their contents during SS production and consumption. According to SS production, the main sources of CML and 5-HMF during SS production are the raw materials used in SS production, the formation of the Maillard reaction during fermentation, and the addition of food additives. In addition, CML and 5-HMF were simultaneously found in commercial LSS, DSS, and infant SS, and the maximum daily intake of CML and 5-HMF could reach 2.40 μg and 120.86 μg, respectively. Also, thermal treatment could increase the CML and 5-HMF contents in commercial SS, which means that daily hot cooking increases our intake of CML and 5-HMF from SS. Fortunately, thermal treatment and the addition of inhibitors could reduce the CML and 5-HMF contents in SS production and daily consumption, respectively. Thermal treatment could simultaneously reduce CML and 5-HMF contents in raw sauce and rude LSS. However, since raw sauce required microorganisms for subsequent fermentation and could not be heat-treated, the thermal treatment performed on rude LSS was much better. Moreover, the addition of EC, EGCG, and VC could reduce or maintain CML and 5-HMF contents during the daily consumption of SS. The addition of an appropriate concentration of EC (100 μM) and VC (5 μM) to SS during direct and heated consumption, respectively, could simultaneously reduce the intake of CML and 5-HMF from SS. This study simultaneously revealed and reduced the CML and 5-HMF contents in SS production and consumption, and provided a theoretical direction for reducing CML and 5-HMF during the production and consumption of heat-processed foods.

## Figures and Tables

**Table 1:** Spearman’s rank correlation coefficients between the change of basic elements and the change in the contents of CML or 5-HMF in the semi-finished sauce.

	Reducing Sugars	Soluble Salt-Free Solids	Protein	Total Nitrogen	FAAs
CML	−0.015	0.576 *	0.015	0.026	0.003
5-HMF	−0.600 **	0.0482	−0.800 **	−0.765 **	−0.841 **

* Significant correlation (*p* < 0.05); ** extremely significant correlation (*p* < 0.01).

## Data Availability

The original contributions presented in the study are included in the article; further inquiries can be directed to the corresponding author.

## Abbreviations

The following abbreviations are used in this manuscript:

SS	Soy sauce
CML	Nε-carboxymethyl-lysine
5-HMF	5-hydroxymethylfurfural
EC	(-)-epicatechin (EC)
EGCG	(-)-epigallocatechin gallate (EGCG)
VC	Ascorbic acid
LSS	Light soy sauce
DSS	Dark soy sauce
FAAs	Free amino acids
